# Vaping during the COVID-19 lockdown period in Belgium

**DOI:** 10.1186/s12889-021-11637-4

**Published:** 2021-09-03

**Authors:** Karolien Adriaens, Dinska Van Gucht, Sven Van Lommel, Frank Baeyens

**Affiliations:** 1 Faculty of Psychology and Educational Sciences, KU Leuven, Tiensestraat 102, 3000 Leuven, Belgium; 2 Thomas More University of Applied Sciences, Molenstraat 8, 2018 Antwerp, Belgium; 3 UZ Leuven, Herestraat 49, 3000 Leuven, Belgium

**Keywords:** Vaping, Electronic cigarettes, COVID-19

## Abstract

**Background:**

Due to the Corona Virus Disease 2019 (COVID-19), the Belgian government set out a range of measures to prevent the spread of the virus. One measure included closing all non-food shops, including vape shops.

**Methods:**

A retrospective online questionnaire was used to investigate the impact of closing the vape shops on the vaping and/or smoking behavior of current vapers.

**Results:**

The sample (*n* = 202) reached consisted of 70% exclusive vapers, 29% dual users and 1% no-product users. Over half (55%, 112/202) of participants needed to buy e-liquid during the lockdown, with a small majority being able to buy e-liquids – mostly with their usual nicotine concentrations, flavor or brand –, but as much as 39% (44/112) of them ran out of e-liquid. Those buying e-liquid mainly did so by making purchases via foreign online webshops. A similar pattern was observed with respect to purchasing hardware, with about half (47%, 95/202) of participants reporting hardware availability and with a small majority (53%, 107/202) reporting hardware unavailability. Of those indicating that hardware was not available, 38% (41/107) ran out of a properly functioning e-cigarette. A non-trivial minority had to consume e-liquids with another nicotine concentration, flavor or brand than usual. One seventh of exclusive vapers before lockdown relapsed partly or completely to smoking during the lockdown. The main reasons for changing vaping and/or smoking behavior included the unavailability of e-liquid with nicotine, the unavailability of hardware, and stress/worries about COVID-19.

**Conclusions:**

The majority of vapers succeeded in maintaining their vaping behavior as usual, highly likely due to (illegally) buying consumables online. Nevertheless, for a minority the lockdown period resulted in unintended consequences and these vapers relapsed (completely) to smoking. Even during periods of lockdown, smokers and vapers should be able to purchase low(er)-risk alternatives to smoking, for example e-cigarettes.

**Supplementary Information:**

The online version contains supplementary material available at 10.1186/s12889-021-11637-4.

## Background

Early March 2020, the first confirmed infections with the Corona Virus Disease 2019 (COVID-19) in Belgium were recorded. In order to prevent the spread of the virus, the government set out a range of measures including closing stores (exceptions: food stores, newsagents and pharmacies) for a period of almost two months (March 18th until May 10th). Consequently, all vape shops were closed and people who vaped (i.e. vapers) could no longer purchase consumables (e.g., e-liquids, batteries, coils) for their electronic cigarettes (e-cigarettes) in brick-and-mortar stores. Some newsagents and some food stores, however, offered a limited product range of e-liquids and e-cigarettes, or temporarily expanded their range (sometimes in cooperation with closed brick-and-mortar stores). Buying consumables online is complicated in Belgium due to the fact that the online sale of e-cigarettes and e-liquids is prohibited [1]. More specifically this prohibition implies that Belgian vape shop owners cannot sell their consumables online (nor do they do so), that foreign webshops cannot sell to Belgian vapers as this is a criminal offence, and that customs can (and occasionally do) seize consumables ordered abroad. The online purchase of consumables itself is not punishable, however. In contrast, people who smoke (i.e. smokers) could still buy their cigarettes in food stores or from newsagents during the lockdown period. Unfortunately, smoking cigarettes is responsible for a very high rate of preventable premature deaths worldwide [2]. The availability of low(er)-risk alternatives to smoking, like e-cigarettes, could be one way to curb the burden of smoking on our society. Currently, it is generally accepted that e-cigarettes are low(er)-risk alternatives to smoking as they are much less harmful to health [3]. E-cigarettes also have the potential to help smokers to quit smoking and to remain smoke-free [4]. The severely restricted availability of low(er)-risk alternatives (i.e. e-cigarettes) during lockdown, however, could have had negative consequences on vaping behavior, including increasing the risk of (re)lapse to smoking.

The purpose of this study was to investigate to what extent the lockdown (i.e. closed vape shops) in 2020 had an impact on vaping and/or smoking behavior among people who were currently vaping (including also dual users of e-cigarettes and tobacco cigarettes). We used a retrospective online questionnaire to investigate the impact of closed vape shops on changes in vaping and/or smoking behavior among current Belgian vapers.

## Methods

### Recruitment

The targeted sample of participants consisted of current (adult + 18 years) Dutch or French speaking Belgian vapers, including exclusive vapers and dual users of e-cigarettes and tobacco cigarettes. In order to recruit participants, advertisements with the link to the online questionnaire were distributed through social media (Twitter, Facebook, “Belgische Damp Bond / Union Belge pour La Vape”). A total of 241 participants started filling out the online questionnaire, with only two (1%) not giving their consent for participation. The questionnaire was fully completed by 202 participants (response rate of 84%).

### Study design

The cross-sectional survey was available in Qualtrics from May 25th until June 8th 2020. After participants clicked on the link, they firstly received general information about the study and were asked to give their consent to participate. When they did not give their consent, participants were thanked for their interest and the questionnaire was finished. Participants who gave their consent, were provided with the questionnaire, thanked at the end and finally were given the opportunity to enter their e-mail address if they wanted to receive information about the results of the study. The majority of the questions were retrospective in nature, meaning that the questions referred to the lockdown period, so from March 18th until May 10th 2020.

The current study is in accordance with the General Data Protection Regulation (GDPR) and was approved by the Societal and Social Ethics Committee of the University of Leuven (G-2020-1960).

### Materials and outcome measures

#### Questionnaire

The questionnaire (see Additional file 1) started with assessing sociodemographic variables like age, gender, highest obtained degree, occupation, marital status, net income per month and nationality (all predefined categories, except age). Next, participants reported on their smoking and vaping status before the lockdown period (i.e. before March 18th 2020) by indicating how frequently (daily, weekly, monthly, not) they were vaping and/or smoking. Subsequently, participants were asked about their stock of consumables (e-liquids and service/hardware), their need to purchase consumables, their ability to purchase these consumables, and how and where they had made such purchases during the lockdown period (i.e. from March 18th until May 10th 2020). This was followed by questions that focused on the type of e-liquid (nicotine concentration) they were using and their vaping frequency (more, less, same as usual, quit vaping) during the lockdown period. Finally, the questionnaire investigated whether any changes had occurred during the lockdown period regarding the vaping and smoking behavior, separately for exclusive vapers and dual users, and what the reasons were for these changes.

#### Statistical analyses

We used descriptive analyses, including frequencies and proportions, to describe the obtained results. Cross-tabulations were used to get a more detailed picture of specific subgroups (e.g., not being able to purchase supplies) and their changes in vaping and/or smoking behavior during the lockdown. Additionally, logistic regressions were carried out to predict smoking initiation among exclusive vapers (starting to smoke vs. remaining smoking abstinent during the lockdown) and to predict a rise in smoking behavior among all participants (smoking more vs. smoking the same/less during the lockdown). Four models were included using three different predictors (i.e. sufficient e-liquid in stock, no vs. yes; ability to purchase e-liquid, no vs. yes; and ability to purchase hardware, no vs. yes). With respect to the ability to purchase e-liquid and hardware, the original response categories (i.e. yes, but not needed due to sufficient stock; yes, and made purchases; no, but not needed due to sufficient stock; no, and fell without) were recoded into simple no vs. yes responses. Only those who explicitly reported that they were not able to purchase e-liquid/hardware and fell without were recoded as those who were not able to make purchases. Some analyses were conducted using Statistica, version 13. Logistic regressions were conducted using SPSS, version 26.0.

## Results

A total of 241 participants started filling out the online questionnaire with 202 participants (response rate of 84%) fully completing the questionnaire. Participants were on average 39 years old (*SD* = 9.89) and were mainly middle-class, full-time working males (all details are presented in Table 1). Before the lockdown period, only one participant (1%) was not smoking nor vaping, 70% of the sample were exclusive vapers (*n* = 142) and 29% were dual users (*n* = 59). Among those dual users, the majority (66%, 39/59) smoked daily and the others smoked weekly (19%, 11/59) or monthly (15%, 9/59). Vapers (including dual users) were vaping daily (96%).

**Table 1:** Sociodemographic characteristics of the total sample (*n* = 202)

Variable	***n***	***M*** (***SD***) or %
**Language**		
Dutch	143	70.79
French	59	29.21
**Age (years)**	202	39.43 (9.89)
**Gender**		
Male	150	74.26
Female	50	24.75
X	1	0.50
Did not wish to answer	1	0.50
**Highest educational degree**		
None	4	1.98
Elementary school	5	2.48
High school	105	51.98
Non-academic bachelor	50	24.75
University	25	12.38
Other	7	3.47
Did not wish to answer	6	2.97
**Occupation**		
Student	7	3.47
Part-time job	7	3.47
Full-time job	153	75.74
Housewife/−man	4	1.98
Job seeker	10	4.95
Invalidity	13	6.44
Retired	4	1.98
Did not wish to answer	4	1.98
**Marital status**		
Single	43	21.29
Relation, not cohabiting	16	7.92
Cohabiting	73	36.14
Married	57	28.22
Divorced	8	3.96
Widow (er)	3	1.49
Other	2	0.99
**Net income per month (in €)**		
< 1000	11	5.45
1000–1500	20	9.90
1501–2000	78	38.61
2001–2500	34	16.83
2501–3000	19	9.41
> 3000	19	9.41
Did not wish to answer	21	10.40
**Nationality**		
Belgian	190	94.06
Other (e.g., Dutch, French)	11	5.45
Missing	1	0.50

**Fig. 1:**
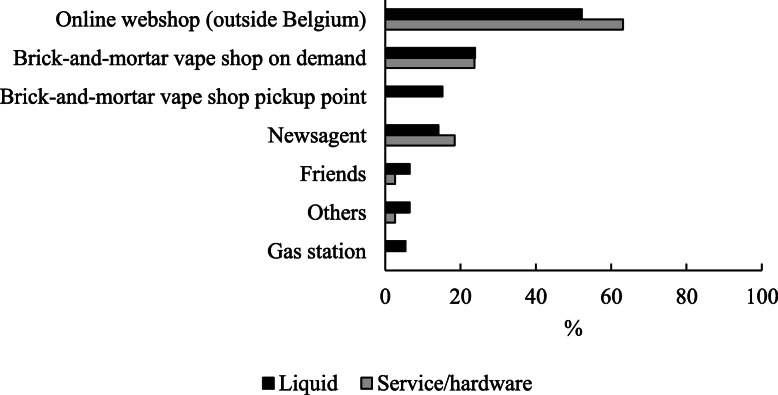
Locations where e-liquid and/or hardware (and service) was purchased. *Note*: E-liquid *n* = 92; Service/hardware *n* = 38

About half (45%, 90/202) of the sample had sufficient e-liquid in stock during the lockdown period, but around one third (31%, 28/90) of them reported buying some extra e-liquid, see Table 2. The other two thirds (69%, 62/90) were not in need of e-liquid nor bought extra e-liquid, but 57% (35/62) of these participants did indicate that they would not have been able to purchase e-liquid if it were needed. In contrast, 55% (112/202) of participants did not have enough e-liquid to bridge the vape shop lockdown period. The majority (57%, 64/112) of them were able to make purchases but 39% (44/112) ran out of e-liquid. The remaining four (4%, 4/112) participants reported not being able to buy e-liquid and that this was not needed. The majority of the participants who did buy e-liquid (*n* = 92) were able to purchase e-liquid with their usual nicotine concentration (89%, 82/92), their usual flavor (65%, 60/92), and of their usual brand (67%, 62/92). Most of them (52%, 48/92) bought e-liquid via an online webshop (outside Belgium), others in a brick-and-mortar vape shop on demand (24%, 22/92), at a brick-and-mortar vape shop pickup point (15%, 14/92), from a newsagent (14%, 13/92), via friends (7%, 6/92) or in a gas station (5%, 5/92), see Fig. 1.

**Table 2:** E-liquid and hardware purchases during lockdown period

Variable	Number	Percent
**Sufficient e-liquid in stock**	**202**	
Yes	90	44.55
No	112	55.45
**Able to purchase e-liquid among total sample**	**202**	
Yes, but not needed due to sufficient stock	27	13.37
Yes, and made purchases	92	45.54
No, but not needed due to sufficient stock	39	19.31
No, and fell without e-liquid	44	21.78
**Able to purchase e-liquid among those with sufficient e-liquid in stock**	**90**	
Yes, but not needed due to sufficient stock	27	30.00
Yes, and made purchases	28	31.11
No, but not needed due to sufficient stock	35	38.89
No, and fell without e-liquid	0	0.00
**Able to purchase e-liquid among those with insufficient e-liquid in stock**	**112**	
Yes, but not needed due to sufficient stock	0	0.00
Yes, and made purchases	64	57.14
No, but not needed due to sufficient stock	4	3.57
No, and fell without e-liquid	44	39.29
**E-liquid bought with nicotine concentration as usual among buyers**	**92**	
Yes	82	89.13
No, needed to buy a higher nicotine concentration	2	2.17
No, needed to buy a lower nicotine concentration	8	8.70
**E-liquid bought with same flavor as usual among buyers**	**92**	
Yes	60	65.22
No, needed to buy another flavor	32	34.78
**E-liquid bought of the same brand as usual among buyers**	**92**	
Yes	62	67.39
No, needed to buy another brand	30	32.61
**Service/hardware available among total sample**	**202**	
Yes, but not needed	57	28.22
Yes, and made purchases	38	18.81
No, but not needed	66	32.67
No, and fell short of a properly functioning e-cigarette	41	20.30

*Note*: % calculated on total sample size or on specified subsamples

We also asked participants to indicate their ability to receive service or purchase any hardware (e.g., e-cigarettes, batteries, coils, atomizers, …) and their need for doing so during the lockdown period (see Table 2). About half of participants (47%, 95/202) indicated that hardware could be purchased, and nearly half (40%, 38/95) of these participants did so. Among the other half of participants (53%, 107/202) who reported that hardware was not available, less than half (38%, 41/107) ran out of properly functioning hardware and the majority (62%, 66/107) were not in need of hardware. Among those who were able to, and did buy some hardware (*n* = 38), the majority (63%, 24/38) did so via an online webshop (outside Belgium) or in a brick-and-mortar vape shop on demand (24%, 9/38) or from a newsagent (18%, 7/38), see Fig. 1.

Regarding changes in vaping and/or smoking behavior during the lockdown period among the total sample, most of the participants (85%, 172/202) consumed e-liquids with the same nicotine concentration (including nicotine-free e-liquids) as usual. A minority switched to using lower nicotine concentrations (7%, 15/202), higher nicotine concentrations (5%, 9/202), or nicotine-free e-liquids (2%, 4/202) compared to their usual nicotine concentrations. Two participants (1%) switched to e-liquid with nicotine whereas they normally vaped nicotine-free e-liquids. The majority of participants reported vaping as much as usual (60%, 121/202). A fourth vaped more than usual (25%, 51/202) and a minority vaped less (8%, 17/202) than usual. Few participants (6%, 13/202) quit vaping during the lockdown period. All participants who indicated that they quit vaping (*n* = 13), reported that they ran out of e-liquid during the lockdown. Most of them (77%, 10/13) also ran out of a properly functioning e-cigarette. Among participants who (temporarily or permanently) ran out of e-liquid (*n* = 44), one third (34%, 15/44) vaped as much as usual, 20% (9/44) vaped more, 16% (7/44) vaped less, and 30% (13/44) quit vaping completely.

Changes in vaping and/or smoking behavior during the lockdown are summarized in Table 3. The majority of exclusive vapers (85%, 121/142) continued with exclusive vaping. A minority combined vaping with smoking tobacco (7%, 10/142) or quit vaping and started smoking tobacco (7%, 10/142), and one participant (1%) quit vaping without starting to smoke tobacco. Among those who indicated that they became a dual user (*n* = 10) during lockdown, the majority (60%, 6/10) ran out of e-liquid, but had sufficient hardware in stock (60%, 6/10). Of those who became exclusive smokers (*n* = 10), all (100%, 10/10) ran out of e-liquid and almost all (90%, 9/10) ran out of a properly functioning e-cigarette. Among dual users (*n* = 59), the majority (68%, 40/59) continued using both products after the lockdown, one fifth (25%, 15/59) became an exclusive vaper, three dual users (5%) quit using both products, and only one participant (2%) became an exclusive smoker (and thus quit vaping), see Table 3. More specifically, the majority of dual users reported smoking more (42%, 25/59) and vaping as much as usual (41%, 24/59), see Table 4 for more details.

**Table 3:** Changes among exclusive vapers and dual users

	Vaping/smoking status after lockdown				
Vaping/smoking status before lockdown	Vaper	Smoker	Dual user	Using no products	Total
**Vaper**	121	10	10	1	142
**Dual user**	15	1	40	3	59
**Using no products**	1	0	0	0	1
**Total**	137	11	50	4	202

*Note*: All are frequencies. No smokers before lockdown period

**Table 4:** Changes in vaping and smoking behavior during lockdown among dual users (n = 59)

	Number	Percent
**Changes in smoking behavior**		
Smoked more	25	42.37
Smoked the same as usual	6	10.17
Smoked less	10	16.95
Quit smoking	18	30.51
**Changes in vaping behavior**		
Vaped more	15	25.42
Vaped the same as usual	24	40.68
Vaped less	16	27.12
Quit vaping	4	6.78

**Fig. 2:**
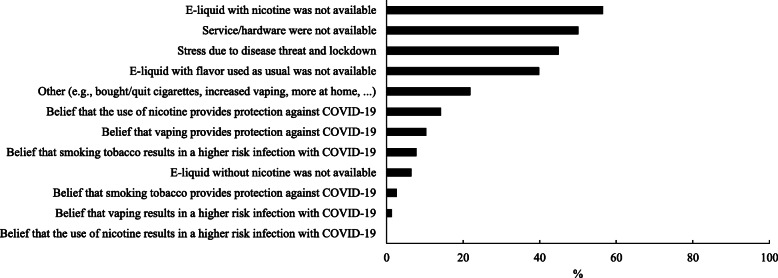
Reasons for changing vaping and/or smoking behavior (*n* = 78)

A majority of participants (61%, 124/202) reported no changes in vaping and/or smoking behavior. Among those that did change their behaviors (39%, 78/202), the main reasons included: e-liquid with nicotine was not available (56%, 44/78), hardware was not available (49%, 38/78), stress and worries about COVID-19 (45%, 35/78), flavor was not available (10%, 31/78), the belief that nicotine provides protection against COVID-19 (14%, 11/78), the belief that the e-cigarette provides protection against COVID-19 (10%, 8/78), the belief that smoking tobacco results in a higher risk infection with COVID-19 (8%, 6/78), and the fact that nicotine-free e-liquid was not available (6%, 5/78), see Fig. 2.

### Logistic regressions

Four logistic regression models were used to predict smoking initiation during the lockdown period among exclusive vapers (see Table 5) and to predict a rise in smoking behavior among all participants (see Table 6). The first model included sufficient e-liquid in stock (no vs. yes) during lockdown as the sole predictor. The odds to start or increase smoking were 22 and 17 times higher, respectively, in participants who did not have sufficient e-liquid in stock than in those who did. Second, when including the sole predictor “ability to purchase e-liquid” (no vs. yes), the odds of starting to smoke vs. remaining smoking abstinent among exclusive vapers, were 77 times higher in participants who were not able to make a purchase than in those who were able to purchase e-liquid. The odds of an increase in smoking behavior were 30 times higher in participants who were not able to buy e-liquid. The third model included the ability to purchase hardware (no vs. yes) as the predictor. The odds to initiate or to increase smoking, were 18 times and eight times higher, respectively, in participants who were not able to purchase hardware than in those who were able to make a purchase. Finally, the fourth model included all three predictors combined from the previous models. With regard to smoking initiation, only one predictor proved to be significant: the odds of smoking initiation were 48 times higher when e-liquid could not be purchased than when it could be purchased. Considering increased smoking behavior as dependent variable, having sufficient e-liquid in stock and the ability to purchase e-liquid were significant predictors. The odds to increase smoking were five and 18 times higher in participants who did not have sufficient e-liquid in stock or were unable to purchase e-liquid, respectively.

**Table 5:** Parameters of logistic regression models for smoking initiation during the lockdown among exclusive users (*n* = 142)

	b (***SE***)	Odds Ratio (95% CI)	R^**2**^ (Nagelkerke)
**Model 1**			.23
Intercept	−4.17 (1.01) ^***^		
E-liquid stock	3.08 (1.04) ^**^	21.67 (2.81, 166.98)	
**Model 2**			.57
Intercept	−3.37 (0.51) ^***^		
E-liquid purchase	4.35 (0.70) ^***^	77.33 (19.67, 303.99)	
**Model 3**			.29
Intercept	−2.54 (0.35) ^***^		
Hardware purchase	2.86 (0.58) ^***^	17.42 (5.59, 54.23)	
**Model 4**			.59
Intercept	−4.17 (1.01) ^***^		
E-liquid stock	1.34 (1.17)	3.84 (0.39, 38.02)	
E-liquid purchase	3.87 (0.98) ^***^	47.77 (7.02, 325.08)	
Hardware purchase	−0.08 (0.96)	0.92 (0.14, 6.04)	

Note: ^**^p < 0.01; ^***^ *p* < 0.001

**Table 6:** Parameters of logistic regression models for an increase in smoking behavior during the lockdown among all participants (*n* = 202)

	b (***SE***)	Odds Ratio (95% CI)	R^**2**^ (Nagelkerke)
**Model 1**			.27
Intercept	−3.37 (0.59) ^***^		
E-liquid stock	2.86 (0.62) ^***^	17.40 (5.17, 58.52)	
**Model 2**			.46
Intercept	−2.41 (0.29) ^***^		
E-liquid purchase	3.39 (0.45) ^***^	29.74 (12.42, 71.21)	
**Model 3**			.21
Intercept	−1.84 (0.23) ^***^		
Hardware purchase	2.09 (0.39) ^***^	8.07 (3.76, 17.32)	
**Model 4**			.50
Intercept	−3.36 (0.59) ^***^		
E-liquid stock	1.62 (0.68) ^*^	5.05 (1.33, 19.15)	
E-liquid purchase	2.88 (0.64) ^***^	17.80 (5.11, 62.01)	
Hardware purchase	−0.22 (0.64)	0.80 (0.23, 2.81)	

Note:^*^p < 0.05; ^***^ *p* < 0.001

## Discussion

One measure taken by the Belgian government to prevent the spread of COVID-19 in 2020 was closing all non-food shops, including the vape shops. The current online retrospective questionnaire study shows that, during this lockdown period, most vapers continued vaping and that even one fifth of dual users became exclusive vapers. Thus, although the brick-and-mortar vape shops were closed and there was a ban on the online sale of e-cigarettes in Belgium, most vapers were able to purchase consumables (e-liquid, hardware) as usual and when needed. Nevertheless, 11, 35 and 33% had to buy e-liquids with a different nicotine concentration, a different flavor, or a different brand than usual, respectively. Although we cannot determine if the vapers reached were already buying consumables online before the lockdown period (as this was not asked), it is not unlikely that this was the case as we made use of a convenience sample of web-savvy vapers. By doing so, it is likely that we reached vapers who were familiar with making online purchases. As the online sale of consumables in Belgium was prohibited, but vapers were still able to make online purchases, this implies two things: 1) that foreign webshops have sold illegally to Belgian vapers while taking the risk to be punished for doing so, and 2) that the ban on the import of consumables purchased online is not enforced.

Being able to purchase consumables online served, for many vapers, as a kind of protective factor against relapse to smoking. If the legislation had been enforced, these vapers might not have been able to obtain consumables and relapse to smoking would probably have been higher. Vapers who are not familiar with buying online or who not prefer to take the risk of buying consumables online, closing vape shops during a lockdown can result in a decrease in the availability of consumables. The limited availability of low(er)-risk alternatives for smoking could consequently result in an increase in smoking behavior. Recent data from the US shows indeed that increasing taxes on e-cigarettes result in an increase in smoking behavior and vice versa [5]. These observations highlight that e-cigarettes act as economic substitutes for regular cigarettes.

It should be stressed that for a non-trivial minority of participants the lockdown period resulted in combining vaping with (more) smoking, or even quitting vaping completely and relapsing to smoking. One seventh of exclusive vapers started smoking (with 60% running out of e-liquid, but having a properly functioning e-cigarette) and or switched completely to exclusive cigarette smoking (with all of them running out of e-liquid and 90% running out of a properly functioning e-cigarette), and the majority of dual users remained using both products while smoking more than usual. Only a minority of dual users switched completely to vaping. Sciensano [6], a federal scientific institution and federal research centre for public health, animal health and food safety in Belgium, also found an increase in smoking prevalence (19.8% in 2018 vs. 21.9% during lockdown 2020). Among smokers, around one third also reported an increase in the amount of cigarettes smoked [6]. The main predictor for smoking initiation or increasing smoking behavior was the inability to purchase e-liquid. Although only a minority of the current sample relapsed to smoking, extrapolation to the Belgian population (approximately 9,500,000 citizens aged 15 year and older) [7] and translating this to absolute numbers may be instructive to estimate the number of vapers that were potentially negatively affected by the lockdown measure in Belgium. If we were to apply our results to the most recent Belgian vaping prevalence data [8], this is what we could expect: According to IPSOS, 10% (950,000 people) of the population vapes at least once in a while, with 3% (285,000 people) vaping daily. Our sample mainly consisted of daily vapers of whom 56% did not have enough e-liquid during the lockdown and of whom 39% ran out of e-liquid. This would amount, in absolute numbers, to 159,600 and 62,244 Belgian daily vapers, respectively. A total of 6% quit vaping, which would translate to 17,100 daily vapers. IPSOS (2019) further reports that 41% of all vapers are exclusive vapers (389,500 people). In our sample, 7% of exclusive vapers relapsed completely to smoking and 7% became a dual user of e-cigarettes and tobacco cigarettes. This would translate to 27,265 people having completely, and the same number having partly, relapsed to smoking. Even though our sample may not be representative for the general (vaping) population, this hypothetical exercise highlights the importance of considering the unintended consequences of closing vape shops and denying access to low(er)-risk products to smokers, while cigarettes remain easily available during lockdown periods.

In contrast to the relapse rates that we observed, Caponnetto and colleagues [9] found that Italian exclusive smokers decreased their cigarette consumption and exclusive vapers showed a similar vaping behavior as before the lockdown. It is not clear what factors could explain these differences. A likely explanation is that Italy is one of the countries that, despite original plans to do so, in the end did not close the vape shops, such that vapers (more) easily could access their consumables during the lockdown period compared to Belgian vapers. There are differences between EU-countries with regard the opening or closing of vape shops during the lockdown periods, for example, vape shops were open in Finland, France, Switzerland, Denmark, Italy, Romania, Norway, Netherlands, Cyprus, and Sweden. In the countries where brick-and-mortar vape shops were closed (UK, Ireland, Spain, and Germany), the online sale of consumables was allowed, in contrast to Belgium. In the longer term, research is needed to investigate the effects of these different regulatory decisions on smoking and/or vaping behavior.

The current results should be read in light of the following limitations. Firstly, we used a convenience sample of current vapers, which may not be representative of the general population of vapers. As a consequence, it can be expected that people who suffered negative consequences of the lockdown on their ability to continue vaping were more likely to participate in the study (i.e. self-selection bias). Secondly, retrospective cross-sectional surveys are prone to recall bias, meaning that participants may experience difficulties to correctly recall specific details about their past behaviors. Thirdly, due to the cross-sectional nature of the study, any potential causal relationships needs to be interpreted with caution. Fourthly, although we distributed the online questionnaire on social media, the response rate was relatively low resulting in a small, mainly male sample. Fifthly, as is true for most questionnaire-based studies, there was no biological verification of self-reported smoking and/or vaping status.

## Conclusions

Carrying out lockdown measures to curb a pandemic can (negatively) impact specific behaviors of specific groups of people. Related to vaping, it is critical to rethink the potential unintended consequences of closing vape shops while there is also a ban on the online sale of e-cigarettes. Smokers and vapers should be provided with the possibility to purchase low(er)-risk alternatives for smoking, even during a lockdown. Denying this opportunity can result in an increase in smoking prevalence, especially when tobacco cigarettes are easily available and the lack of available e-liquids/hardware, and exposure to toxic substances due to the lack of maintenance of e-cigarettes.

## Supplementary Information

**Additional file 1.** Online questionnaire. The additional file contains the online questionnaire translated into English.

## Data Availability

The datasets used and/or analyzed during the current study are available from the corresponding author on reasonable request.

## Abbreviations

COVID-19: Corona Virus Disease 2019
E-cigarette: Electronic cigarette
