# Supplementary material for: Vaping during the COVID-19 lockdown period in Belgium
Source: BMC Public Health. 2021 Sep 3;21:1613. doi: 10.1186/s12889-021-11637-4 (PMC8414467; doi:10.1186/s12889-021-11637-4)
Supplement: Supplementary file 1 — Additional file 1. Online questionnaire. The additional file contains the online questionnaire translated into English. [file 12889_2021_11637_MOESM1_ESM.docx]

**Additional File 1 – Online questionnaire**

*The following questions were presented to all participants:*

The following questions ask for some general information. Please fill out the questions as truthfully as possible.

1. Age (in years): …
2. Gender:
   1. Male
   2. Female
   3. X
   4. I do not wish to answer
3. Highest educational degree:
   1. None
   2. Elementary school
   3. High school
   4. Non-academic bachelor
   5. University
   6. Other: …
   7. I do not wish to answer
4. Occupation:
   1. Student
   2. Part-time job
   3. Full-time job
   4. Housewife/-man
   5. Job seeker
   6. Invalidity
   7. Retired
   8. I do not wish to answer
5. Marital status:
   1. Single
   2. Relation, not cohabiting
   3. Cohabiting
   4. Married
   5. Divorced
   6. Widow(er)
   7. Other: …
6. Net income per month (in €):
   1. < 1000€
   2. 1000-1500
   3. 1501-2000
   4. 2001-2500
   5. 2501-3000
   6. > 3000
   7. I do not wish to answer
7. Nationality:
   1. Belgian
   2. Other: …
8. For each statement, indicate what best describes your smoking and/or vaping behavior during the period before the lockdown (i.e., before 18/03/2020):

|  | Daily | Weekly, but not daily | Monthly, but not weekly | Not |
| --- | --- | --- | --- | --- |
| I smoked … |  |  |  |  |
| I vaped … |  |  |  |  |

Each of the following questions concerns the lockdown period in which the vape shops were closed (18/03/2020 up to and including 10/05/2020), unless otherwise described. Please fill out the questions as truthfully as possible.

1. During the lockdown period, did you have a sufficiently large stock of e-liquid, so that you did not have to buy new one?
   1. Yes, I had enough e-liquid in stock to bridge the lockdown period.
   2. No, I had insufficient e-liquid in stock to bridge the lockdown period.
2. Have you been able to purchase e-liquid during the lockdown period?
   1. Yes, but that was not necessary because I had sufficient stock.
   2. Yes, and I bought some as well.
   3. No, I could not buy e-liquid, but that was not necessary because I had sufficient stock.
   4. No, I could not buy e-liquid, so I ran out of it.

*The following questions were only presented to participants who answered question 10 with option b:*

1. When you have purchased e-liquid during the lockdown period, have you been able to buy e-liquid with the nicotine dose (mg/mL) you generally use and prefer?
   1. Yes.
   2. No, I had to buy e-liquid with a higher dose (mg/mL).
   3. No, I had to buy e-liquid with a lower dose (mg/mL).
2. If you have purchased e-liquid during the lockdown period, have you been able to buy e-liquid with the flavor you generally use and prefer?
   1. Yes.
   2. No, I had to buy e-liquid with another flavor.
3. If you have purchased e-liquid during the lockdown period, have you been able to buy e-liquid of the brand you generally use and prefer?
   1. Yes.
   2. No, I had to buy e-liquid of another brand.
4. If you have purchased e-liquid during the lockdown period, where did you make your purchases? (multiple answers are possible)
   1. At a brick-and-mortar vape shop (on demand)
   2. At a brick-and-mortar vape shop pickup point (e.g., at newsagent)
   3. At a newsagent
   4. At a gas station
   5. Via an online webshop (outside Belgium)
   6. Via friends
   7. Others: …

*The following questions were presented to all participants:*

1. Were parts/service (purchase new device, coils, battery, clearomizer, charger, repair) available during the lockdown period?
   1. Yes, but that was not necessary, I had enough well-functioning materials in stock.
   2. Yes, and I also made use of that service (bought coils/battery/clearomizer).
   3. No, but I did not need any hardware/service either, because I had enough materials in stock.
   4. No, and so I no longer had a properly functioning e-cigarette.

*The following questions were only presented to participants who answered question 15 with option b:*

1. When you have purchased hardware or needed service during the lockdown period, where did you find hardware/service (new e-cigarette, coils, battery, clearomizer, charger, repair)? (multiple answers are possible)
   1. At a brick-and-mortar vape shop (on demand)
   2. At a brick-and-mortar vape shop pickup point (e.g., at newsagent)
   3. At a newsagent
   4. At a gas station
   5. Via an online webshop (outside Belgium)
   6. Via friends
   7. Others: …

*The following questions were presented to all participants:*

1. What type of e-liquid (with/without nicotine) did you use during the lockdown period?
   1. I used nicotine free e-liquid, as usual.
   2. I used nicotine-free e-liquid, but normally I use e-liquid containing nicotine.
   3. I used nicotine-containing e-liquid, but with a lower nicotine dose (mg/mL) than usual.
   4. I used nicotine-containing e-liquid, but with a higher nicotine dose (mg/mL) than usual.
   5. I used nicotine-containing e-liquid, with the same nicotine dose (mg/mL) as usual.
   6. I used nicotine-containing e-liquid, but normally I use nicotine-free e-liquid.
2. What was your vape frequency during the lockdown period?
   1. I vaped more (e.g., at more moments, for longer periods, more e-liquid consumed).
   2. I vaped less (e.g., at fewer moments, for shorter periods, less e-liquid consumed).
   3. I did not vape more nor less, I vaped as just as much as usual.
   4. I have quit vaping.

*This question was only presented to participants who were exclusive vapers before the lockdown.*

1. If you only vaped and hence did not smoke before the lockdown, what changes have occurred to your vaping behavior and possible smoking behavior during the lockdown period?
   1. I still only used an e-cigarette.
   2. I used an e-cigarette + another form of smokeless nicotine (NRT like patches, Snus).
   3. I used an e-cigarette + I smoked tobacco.
   4. I quitted using an e-cigarette + I smoked tobacco.
   5. I quitted using an e-cigarette (I did not use anything anymore).

*This question was only presented to participants who were dual users before the lockdown.*

1. If you vaped as well as smoked before the lockdown, what changes have occurred to your vaping and smoking behavior during the lockdown period?

|  | more | as much | less | not anymore |
| --- | --- | --- | --- | --- |
| During the lockdown I smoked … tobacco than before the lockdown. |  |  |  |  |
| During the lockdown I used an e-cigarette … than before the lockdown. |  |  |  |  |

*This question was only presented to participants who were exclusive smokers before the lockdown*

1. If you only smoked (and hence did not vape) before the lockdown, what changes have occurred to your vaping and smoking behavior during the lockdown period?
   1. I smoked tobacco + used an e-cigarette.
   2. I did not smoke tobacco anymore + only used an e-cigarette.

*The following question was presented to all participants:*

1. If you have adjusted your vaping and/or smoking behavior, please indicate which of the following reasons best describe the reasons why (multiple answers are possible):
   1. I have not changed my vaping and/or smoking behavior.
   2. The e-liquid with nicotine was not available.
   3. The e-liquid without nicotine was not available.
   4. The e-liquid with the flavor I normally use was not available.
   5. Hardware/service was not available.
   6. Stress due to threat of illness and lockdown.
   7. The belief that smoking tobacco results in quicker infection and more complications due to COVID-19.
   8. The belief that using an e-cigarette results in quicker infection and more complications due to COVID-19.
   9. The belief that using nicotine results in a quicker infection and more complications due to COVID-19.
   10. The belief that smoking tobacco results in protection against COVID-19.
   11. The belief that using an e-cigarette results in protection against COVID-19.
   12. The belief that using nicotine results in protection against COVID-19.
